# Characterization, Antibacterial Evaluation and Computational Study of Synthesized 4,5‐bis(Hydroxymethyl)‐2‐Methylpyridin‐3‐ol Tetraphenylborate Ion‐Pair Complex

**DOI:** 10.1002/open.202400422

**Published:** 2025-02-10

**Authors:** Ahmed H. Bakheit, Mohamed H. Al‐Agamy, Rashad Al‐Salahi, Essam Ali, Haitham Alrabiah, Gamal A.E. Mostafa

**Affiliations:** ^1^ Department of Pharmaceutical Chemistry College of Pharmacy King Saud University P.O. Box 2457 Riyadh 11451 Saudi Arabia; ^2^ Department of Pharmaceutics College of Pharmacy King Saud University P.O. Box 2457 Riyadh 11451 Saudi Arabia

**Keywords:** Pyridoxine, Ion-pair complex, Characterization, Antibacterial activity, Computational study, Quantum Theory of Atoms in Molecules (QTAIM), Reduced density gradient (RDG) analysis, Non-covalent interaction (NCI)

## Abstract

The synthesis of 4,5‐bis(hydroxymethyl)‐2‐methylpyridin‐3‐ol tetraphenylborate complex in water using an anion exchange process yielded more than 76 %. The resulting white complex was obtained and characterized using various spectroscopic and analytical techniques, including ultraviolet, infrared radiation (IR), mass, elemental analysis, and nuclear magnetic resonance (NMR). The antimicrobial activity of the formed ion‐associate complex was evaluated. The structural, electrical, and bonding properties of a novel pyridoxine‐tetraphenylborate ion‐pair complex was explored using B3LYP/6‐311G(d,p) DFT simulations. Geometries designed for negative complexation energy showed thermodynamically beneficial complex formation. Reduced density gradient (RDG) analysis and non‐covalent interaction (NCI) plots showed that van der Waals forces are essential to complex stability. Quantum Theory of Atoms in Molecules (QTAIM) study detected weak and moderate hydrogen bonds in the complex using bond critical point (BCP) features. These results reveal how molecules form and stabilize the pyridoxine‐tetraphenylborate ion‐pair complex. To know the interaction between receptors and bioactive chemicals, one must understand the mechanism of the ionic complexes formed between bioactive chemicals and/or organic molecules.

## Graphical abstract

A novel 5‐bis(hydroxymethyl)‐2‐methylpyridin‐3‐ol tetraphenylborate complex was synthesized. The structure was characterized using various spectroscopic techniques. The complex exhibited notable antifungal activity and effectiveness against Gram‐positive bacteria. Theoretical and experimental studies confirmed intermolecular interactions. Computational analyses revealed detailed structural and electrical properties.

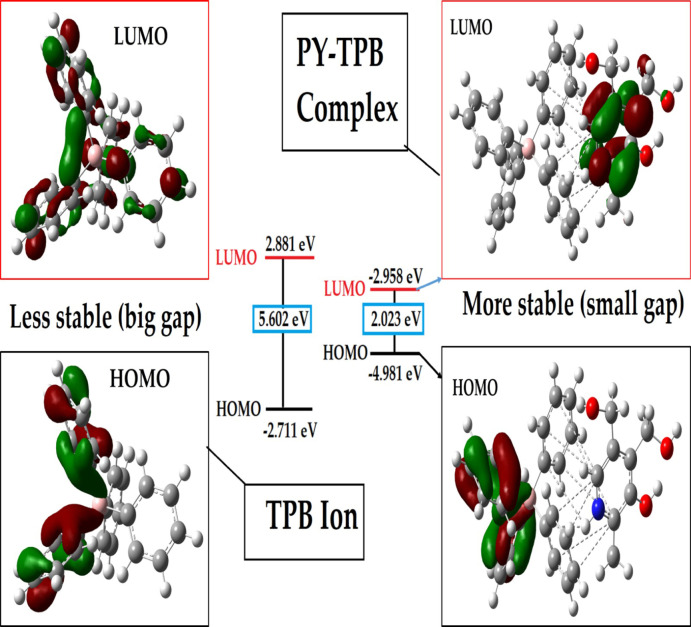

## 1 Introduction

Pyridoxine (Vitamin B6) is a water‐soluble B vitamin essential to nervous system function.^[1]^ Skin and immune systems need VB6. Some enzymes that break down proteins, amino acids, and other substances require VB6.^[2]^ VB6 support human antibody production, brain signal transmission, hemoglobin production, blood glucose regulation, and immune system maintenance.^[3]^ VB6 deficiency can cause dementia, impaired alertness, convulsions, irritability, autonomic dysfunction, and cognitive impairment in adults. In children, VB6 deficiency can affect the CNS.^[4]^

Pyridoxine, pyridoxamine, and pyridoxal are vitamin B6 molecules that change ionic states with pH and temperature.^[5]^ Pyridoxine is usually a cation in acidic solutions. It is an anion in alkaline conditions and a mixture of unionized and zwitterionic forms in neutral settings. These ions have unique spectra. Pyridoxal phosphate reacts better with aldehydes than free pyridoxal, which is stable in pure solutions but not in nature. Animal tissues can convert all three forms into active derivatives like pyridoxal and pyridoxamine phosphate.

Pharmaceutical chemicals are weak acids or bases; thus, salts improve their behavior in aqueous solutions. When cations and anions in these salts interact, complexes or ion pairs develop. These contain two or more ions close together with hydrocarbon groups hiding their charges. This unique structure makes it less water‐loving than the ions alone, making it simpler to dissolve in non‐polar solvents and separate into lipid phases.^[6]^ Pyridoxine hydrochloride dissolves well in water but has a poor partition coefficient (log P=−4.32), making it hydrophobic even in salt form.

Early studies show that pyridoxine can combine with isoniazid and nicotinic acid to make eutectics. It can also combine with para‐aminobenzoic acid and saccharin to make molecular salts^[7]^. Studies have made and tested 23 new bis‐phosphonium salts that come from pyridoxine to see how well they kill bacteria.^[8]^ Furthermore, studies have demonstrated the interaction of pyridoxine hydrochloride with surfactants such as sodium cholate and hexadecyltrimethylammonium bromide, indicating its potential application in diverse physicochemical contexts.^[9]^

Ion‐pair complexes find utility in diverse disciplines in the biological, physical, environmental, and chemical sciences. Their unique properties make ion‐pair complexes useful in many disciplines.^[10]^ Analytical chemistry uses the ion‐pair complex to stabilize the chromophore to detect lower concentrations of certain ions.^[10a,c]^ Controlled release formulations improve pharmacokinetics and pharmacodynamics, while ion‐pairing stabilizes and dissolves medicines.^[6]^ Protein purification with ion pairs affects nucleic acid‐protein interactions in biochemistry.^[11]^ Ion‐pair complexes detect and assess heavy metals and anionic pollutants in water and soil samples in environmental chemistry.^[12]^ Ion‐pair complexes in material sciences create conducting polymers that improve electrical conductivity or physical characteristics for electronic applications.^[13]^ Intermediate ion pairs can stabilize reactive species or transition states in organic chemistry catalysis.^[14]^ Developing sensitivity‐enhancing electrochemical sensors that detect specific ions or chemicals requires ion‐pair complexes.^[15]^ As an example, these uses include sensing and identifying things, making machines that are mechanically interlocked, changing the behavior of ion pairs by adding photoswitchable parts, and changing behavior by changing non‐covalent interactions like cation‐π, anion‐π, CH‐π, hydrogen bonding, and halogen bonding.^[16]^

The goal of this research is to synthesize, characterize, and computational investigate the pyridoxine‐tetraphenyl borate ion‐associate complex. This study is based on ion‐pair complex systems, which many properties including receptor activity, recognition behavior, photoswitchability, extraction, and transport capabilities. The relevance of these ion‐pair complex systems serves as the motivation for this study. Conversely, we conduct an assay to evaluate the biological activity of the complex under investigation, particularly its antibacterial activity.

## Experimental

### Materials and Instrument

All the chemicals and reagents were analytical reagent grade. Pyridoxine hydrochloride (purity >99.5) was purchased from Sigma‐Aldrich (Darmstadt, Germany), and sodium tetraphenyl borate was purchased from Merck (Darmstadt, Germany).

The melting point was determined using the Gallenkamp melting point instrument. NMR spectra were scanned in DMSO‐d6 using a Brucker NMR spectrometer at 500 MHz for ^1^H and 125 MHz for ^13^C. Chemical shifts are measured in δ‐values (ppm) relative to TMS, an internal standard. Coupling constants (*J*) are stated in Hertz. D_2_O was used to confirm the exchangeable protons. The mass spectrum was obtained using an Agilent Triple Quadrupole 6410 QQQ LC/MS with an electrospray ionization (ESI) source. A Shimadzu 1800 UV double beam spectrophotometer with quartz cell was employed, as well as a PerkinElmer PE 2400 series II CHNS/O analyzer for elemental analysis. The X‐ray powder diffraction (XRD) pattern was performed using a Simmon XRD‐5000 diffractometer.

### Synthesis of 4,5‐bis(Hydroxymethyl)‐2‐Methylpyridin‐3‐ol Tetraphenylborate

To a solution of 4,5‐bis(hydroxymethyl)‐2‐methylpyridin‐3‐ol;hydrochloride (pyridoxine hydrochloride) (0.206 g, 1 mmol) in deionized water (25 mL), a solution of sodium tetraphenylborate (0.3422 g, 1 mmol) in deionized water (25 mL) was added. The white precipitate was filtered, washed with cold deionized water, and dried over anhydrous CaCl₂, yielding over 75 %. The melting point of the ion‐associate complex was 167 °C, which is different than the reactants (207 °C and >310 °C) for pyridoxine and sodium tetraphenylborate, respectively. IR (KBr cm^−1^): 3185–3500 cm^−1^ for OH and 1257 cm^−1^ for C−N. ^1^H NMR (700 MHz, DMSO_d_6_) δ: 2.4 (s, CH_3_, 3H), 4.29 (s, CH_2_, 2H), 4.54 (s, OH, 2H), 4.78 (s, CH_2_, 2H), 66.86–7.15 (all Ar−H), 8.03 (s, 1H, pyridine H). ^13^C NMR (175 MHz, DMSO‐d_6_) δ: 15.36 (CH_3_), 56.64 (−CH_2_−), 58.13 (−CH_2_−), 122.34, 125.98, 126.96, 128.86, 130.04, 133.22, 135.94, 137.90, 140.27, 142.48, 152.45, 163.56, 163.83, 164.21.

### Antimicrobial Activity

The antibacterial activity of the ion‐pair complex was tested against four standard bacterial strains (*Staphylococcus aureus* ATCC 25923, *Bacillus subtilis* ATCC 10400, *Escherichia coli* ATCC 25922, and *Pseudomonas aeruginosa* ATCC 27853) as well as one yeast fungus strain (*Candida albicans* ATCC 90028). The cup diffusion method was used to screen for antimicrobial activity, and the minimum inhibitory concentrations (MICs) were determined.

#### Cup Diffusion Method

The cup diffusion method was done in accordance with CLSI rules.^[18]^ We streaked the usual strains over non‐inhibitory tryptone soy agar. The plates were incubated aerobically at 37 °C for 20 hours. Pure colonies of fresh culture were picked up with inoculating loops and suspended in three milliliters of sterile phosphate buffered saline. The suspension was properly blended using a vortex mixer. We calibrated the suspension spectrophotometrically at a wavelength of 600 nm. The amended inoculum was diluted using CLSI criteria, resulting in a final stock suspension of 1×10^6^ colony forming units (CFU)/mL. The sterile cotton swab was dipped in the suspension and turned against the side of the tube. Swabbed the bacterial suspension evenly across the Mueller‐Hinton agar (MHA) plate and swabbed *C. albica*n*s* suspension evenly across MHA plate contained 2 % glucose and 0.5 μg/mL methylene blue. Cups were formed on the inoculated plates using a cork borer. Using a pipette, 100 μL of DMSO containing 100 μg of complex was added to the cup of the inoculated MHA plates. Ciprofloxacin disc and fluconazole disc served as antibacterial and antifungal quality standard controls, respectively. The plates were incubated aerobically at 37° degrees Celsius for 20 hours. After incubation, the data were recorded by measuring the zone of inhibition (mm) with a ruler. The cup diffusion procedure was performed in triplicate, and the average value was calculated.

#### Minimum Inhibitory Concentration (MIC)

The MIC approach followed the EUCAST criteria.^[17]^ The MIC was calculated using the microbroth dilution method using standard ATCC strains that had previously been utilized in the cup diffusion method. An accurate amount of 50.12 mg of the proposed complex was dissolved in DMSO to yield a stock solution of 50.12 mg/mL. The appropriate amount of stock was diluted 1 : 10 in both media to produce a working solution of 5.012 mg/mL. A sterile 96‐microtiter plate (12×8 wells) was employed. Each row (12 wells) was utilized to calculate the MIC of the substance per organism. Each well received 100 μL of sterile Mueller‐Hinton broth for bacterial strains and RPMI 1640 (with L‐glutamine and a pH indicator but no bicarbonate) supplemented with glucose to a final concentration of 2 % for yeast strains. The first ten wells received a two‐fold serial dilution. The working solution (5.12 mg/mL) was diluted by adding 100 μL to the first well and thoroughly mixing. Next, 100 μL of the first well was pipetted into the second well and carefully mixed. The technique was repeated until the tenth well, after which 100 μL was discarded. 100 μL of the adjusted inoculum (1×10^6^ CFU/mL) was added from the first to tenth wells. The eleventh and twelfth wells served as negative and positive controls, respectively. Ciprofloxacin and fluconazole served as antibacterial and antifungal positive controls, respectively. The microtiter plate was incubated at 37 °C for 20 hours. After the incubation time, the MIC values were manually recorded.

### Computational Details

Density Functional Theory (DFT) has become a cornerstone in computational quantum chemistry over the past few decades.^[19]^ Conformational geometries of the target molecule were determined using DFT, with all calculations performed using the Gaussian 09 software package.^[20]^ The B3LYP functional,^[21]^ combined with the 6‐311G(d,p) basis set,^[22]^ was employed due to its balance of accuracy and computational cost. Initial geometries were derived using traditional geometrical parameters and optimized at the DFT level without constraints on the potential energy surface. Time‐dependent DFT (TD‐DFT) was utilized to determine electronic absorption spectra based on the optimized structure.^[23]^

For the development of fundamental structures of complex ion pairs, geometries of Pyridoxine (PY), tetraphenylborate (TPB), and their salt were used. These interactions resulted in various ion pair configurations, and one of these geometries was used for vibrational frequency computations to ensure all stationary points were true minima (no imaginary frequencies) and to evaluate their thermodynamic properties.^[24]^

Proton affinities of Pyridoxine and its conjugate bases were estimated by calculating the difference in enthalpy values between the cations and their respective acids. The Gibbs free energy change (ΔG_298_) for ion pair formation was computed by comparing the free energy of the ion pair to the sum of the free energies of the individual ions.^[25]^ Interaction energy (E_int_) between the ions in the pair was calculated using the supermolecule approach and corrected for basis set superposition error (BSSE) using the Boys and Bernardi counterpoise method.^[26]^

The Quantum Theory of Atoms in Molecules (QTAIM) approach was used to analyze the topological properties of the electron density.^[27]^ This analysis focused on the bond critical points (BCPs) and the bond paths, which are indicative of chemical bonding and interaction strength. Key metrics such as the electron density (ρ(r)) at the BCP, its Laplacian (∇^2^ρ(r)), and the total energy density (H(r)) were computed to characterize bonding interactions.^[28]^ The AIMAll software was used for these QTAIM calculations.^[29]^

Global and local indices were computed using the Koopman method.^[30]^ Single‐point calculations of the anion and cation at the optimized geometry of the neutral molecule were performed to obtain these indices, maintaining a constant external potential. The molecular electrostatic potential (MEP) surface was visualized using GaussView.^[31]^ Additionally, Mulliken population analysis was conducted to determine the single‐point energies of the neutral, cationic, and anionic species using the 6‐311G(d,p) basis set. All DFT calculations were carried out at the ground state energy level, ensuring no constraints on the potential energy surface.

## 2 Results and Discussion

### 2.1 Chemistry

**Scheme 1:**
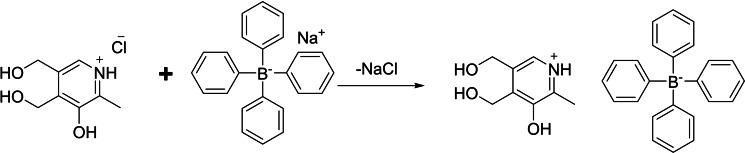
Reaction of pyridoxine HCl with sodium tetraphenyl borate.

The proposed compound of a 4,5‐bis(hydroxymethyl)‐2‐methylpyridin‐3‐ol tetraphenylborate, was synthesized with a yield of 75 % from the reactions between sodium tetraphenyl borate and 4,5‐bis(hydroxymethyl)‐2‐methylpyridin‐3‐ol hydrochloride (pyridoxine hydrochloride) in deionized water at room temperature (Scheme 1). Electrospray ionization mass spectrometry (ESI‐MS) revealed a peak at (m/z) 170 for [M]^+^, in addition to a negative scan at (m/z) 319.2 for [M^−^] (Figure S1). In accordance with the theoretical values of C 78.62 %, H 6.34 %, and N 2.86 %, the elemental analysis data reveal C 77.35 %, H 6.59 %, and N 2.84 %.

The ^1^H NMR spectrum of the pyridoxine‐tetraphenyl borate complex revealed a singlet signal for the CH_3_ proton at 2.4 ppm, as well as signals for the CH_2_ proton at 4.29 ppm and t 4.78 ppm. The OH proton at 4.54 ppm, and the pyridine ring proton at 8.03 ppm. The aromatic protons of the tetraphenyl moiety were detected in the range of 6.86 to 7.15 ppm (Figure S2). The ^13^C NMR spectrum of the target complex, resonance peaks at 15.36 ppm for the CH_3_ group, 56.64 and 58.13 ppm allocated for both CH_2_ carbons, and aromatic carbons in the pyridine and tetraphenyl rings were found between 122.34 and 164.21 ppm (Figure S3).

Additionally, the infrared spectrum of the complex exhibited a C−N stretching vibration peak was shown at 1257 cm^−1^ in the infrared spectrum of the target complex, and the absorption bands at 3185–3500 cm^−1^ were attributed to hydroxyl groups (Figure S4).

The XRD pattern of the 4,5‐bis(hydroxymethyl)‐2‐methylpyridin‐3‐ol tetraphenylborate complex reveals a crystalline nature. This is evident from the presence of sharp, distinct peaks in the pattern. Amorphous materials, in contrast, typically exhibit broad and diffuse peaks.

**Figure 1:**
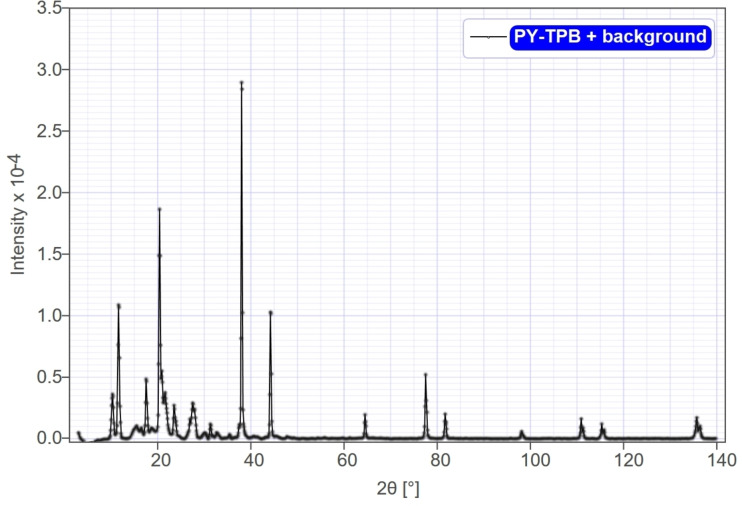
XRD pattern confirms the crystalline nature of 4,5‐bis(hydroxymethyl)‐2‐methylpyridin‐3‐ol tetraphenylborate complex with distinct diffraction peaks.

The positions of these peaks are intricately linked to the interplanar distances inside the crystal lattice of the complex, indicating the excellent purity of the proposed complex. Higher intensity peaks correspond to planes with greater atomic or electron densities, as shown in Table S1. The intensities of the peaks reflect the abundance of different crystal planes in the sample (Figure 1).

### 2.2 Antimicrobial Activity

#### 2.2.1 Cup Diffusion Method

Table 1 shows the results of the cup diffusion test. The cup diffusion test demonstrated good antibacterial activity against tested gram‐positive bacteria (*S. aureus* ATCC 25923 and *B. subtilis* ATCC 10400) and antifungal activity against *C. albicans* ATCC 90028. However, the compounds had weak antibacterial activity against gram‐negative bacteria (*E. coli* ATCC 25922 and *P. aeruginosa* ATCC 27853).

**Table 1:** Inhibition zones and MIC values of the chemical compound against bacteria and yeast.

Compound	*S. aureus* ATCC 25923		*B. subtilis* ATCC 10400		*E. coli* ATCC 25922		*P. aeruginosa* ATCC 27853		*C. albicans* ATCC 10231	
	Inhibition zone (mm)	MIC (μg/ml)	Inhibition zone (mm)	MIC (μg/ml)	Inhibition zone (mm)	MIC (μg/ml)	Inhibition zone (mm)	MIC (μg/ml)	Inhibition zone (mm)	MIC (μg/ml)
Ion‐associate	30	16	27	32	8	ND	10	ND	30	16
Ciprofloxacin	34	0.25	30	≤0.25	32	≤0.25	29	0.25	ND	ND
Fluconazole	ND	ND	ND	ND	ND	ND	ND	ND	32	2
DMSO	NIZ	ND	NIZ	ND	NIZ	ND	NIZ	ND	NIZ	ND

ND= Not determined, NIZ=No inhibition zone.

#### 2.2.2 Minimum Inhibitory Concentration (MIC)

The MIC was determined using the quality control ATCC strains (*S. aureus* ATCC 25923, *B. subtilis* ATCC 10400, and *C. albicans* ATCC 90028). The MIC results are reported in Table 1. The MIC values are 8 μg/mL, 16 μg/mL, and 16 μg/mL for *S. aureus* ATCC 25923, *B. subtilis* ATCC 10400, and *C. albicans* ATCC 90028, respectively. Cup diffusion and MIC results indicated that the ion‐associate complex was effective against gram‐positive bacteria and yeast.

### 2.3 Computational Study

#### 2.3.1 Geometrical Optimization Using DFT Calculation

**Figure 2:**
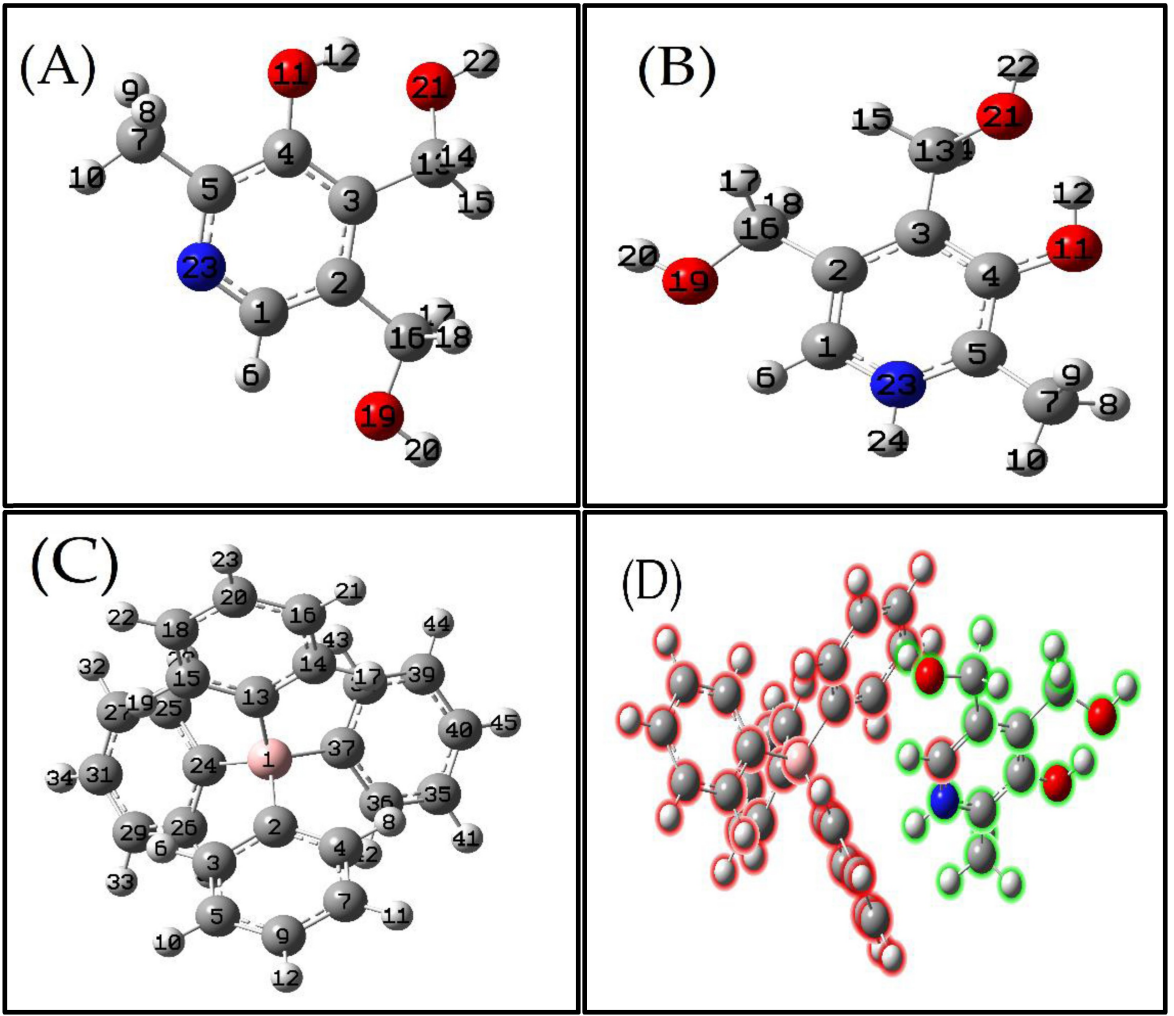
Optimized geometries obtained using B3LYP/6‐311G(d,p): (A) Pyridoxine (PY), (B) Pyridoxine cation (PY cation), (C) Tetraphenylborate anion (TPB anion), (D) Pyridoxine (green)‐tetraphenylborate salt (red) (PY‐TPB salt).

For the optimization of the geometries of pyridoxine cation, pyridoxine tetraphenylborate anion, and the pyridoxine‐tetraphenylborate salt in the gas phase, the B3LYP density functional theory with the 6–311 G(d,p) basis set was identified as the most suitable method (Figure 2).^[32]^

Due to the unavailability of the crystal structure of the compound, it was not possible to compare the calculated bond lengths and angles with experimental data. Figure 2 illustrates the geometries that best represent the energetically optimal ion pair configurations, highlighting their thermodynamic stability and bonding interactions. Specifically, the optimized structure of the pyridoxine (Figure 2A) pyridoxine cation (Figure 2B), while the optimized structure of the tetraphenylborate anion (Figure 2C). Furthermore, the optimized geometries of the ion pair salt are presented in (Figure 2D). All these geometries correspond to local minima on the potential energy surface.

#### 2.3.2 Interaction Energies (IE)

The Density Functional Theory (DFT) method, employing the B3LYP hybrid functional^[33]^ and the 6‐311G(d,p) basis set,^[22]^ is widely recognized for its effectiveness in determining the most stable configurations and binding energies of molecular structures.^[34]^ Accordingly, the complexation energies and Basis Set Superposition Error (BSSE) corrected energies^[26]^ for the pyridoxine (PY) cation, tetraphenylborate (TPB) anion, and the pyridoxine‐tetraphenylborate salt (PY‐TPB) at a 1 : 1 molar ratio were calculated and are presented in Table 2.

**Table 2:** Interaction energies with the (ΔE) _corrected_ and ΔEBSSE, respectively in kcal mol^−1^ at the B3LYP /6‐311G(d,p) level of theory.

Complexes	(ΔE) raw (kcal/mol)	(ΔE) corrected (kcal/mol)	ΔEBSSE
PY– TPB	−65.16	−62.28	0.00459

In the gas phase, the pyridoxine‐tetraphenylborate salt exhibits a complexation energy of −62.28 kcal/mol and a BSSE‐corrected energy of 0.00459 kcal/mol. These findings indicate low formation energies, suggesting that the complexes are highly stable. Furthermore, the negative complexation energies imply that the formation of these complexes is thermodynamically favorable, consistent with experimental observations.^[35]^

#### 2.3.3 NCl‐RDG Analysis

**Figure 3:**
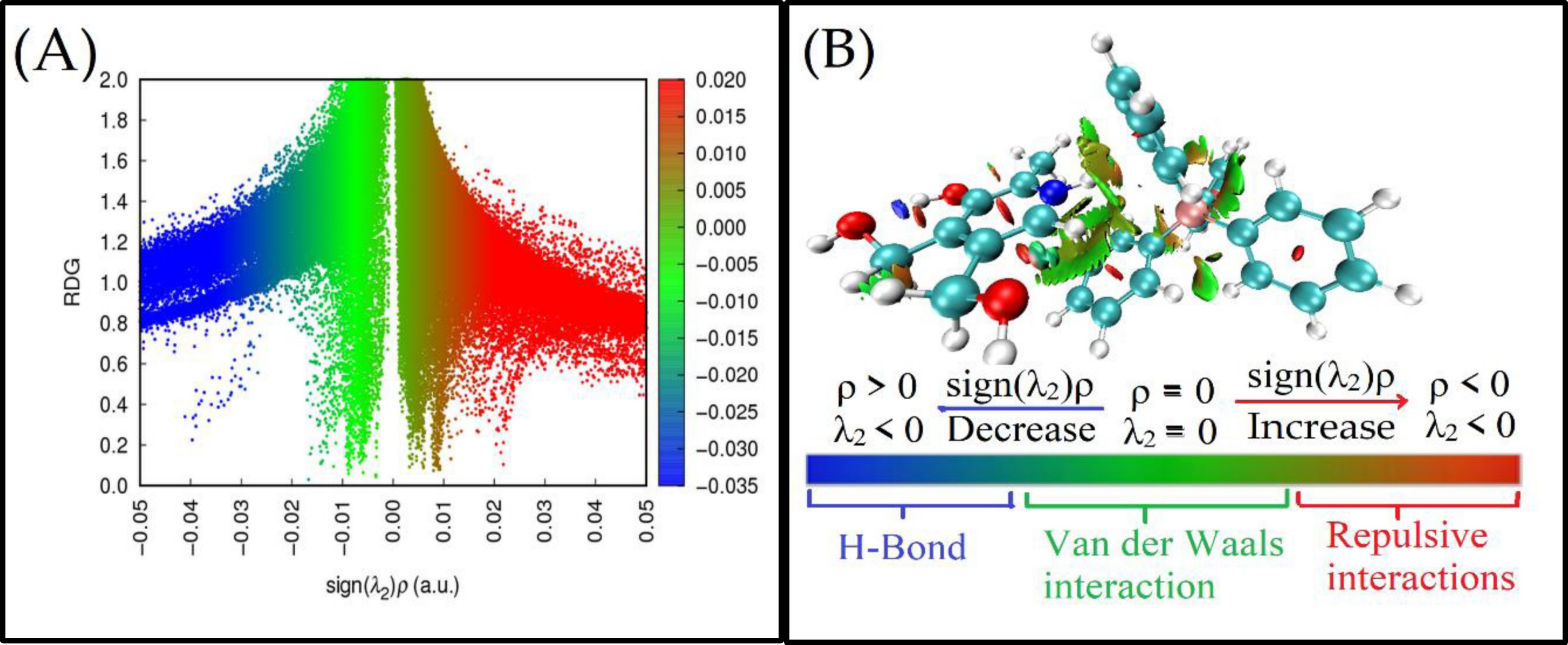
Standard Non‐Covalent Interaction (NCI) index representations and Reduced Density Gradient (RDG) scatter plots (A&B) at the B3LYP/6‐311G level. The RDG cut‐off is set at sign(λ_2_)ρ=0.5 a.u., with a color scale ranging from −0.030 to 0.02 a.u. Blue, green, and red surfaces indicate attractive, van der Waals, and repulsive interactions, respectively, for PY‐TPB configurations.

Similar to the Atoms in Molecules (AIM) approach, the Reduced Density Gradient (RDG) methodology is an effective tool for analyzing non‐covalent interactions. RDG scatter plots and Non‐Covalent Interaction (NCI) plots can be employed to visualize these interactions between molecule ions.[^35^, ^36^] In this technique, RDG is plotted against the electron density multiplied by the sign of the second eigenvalue (sign(λ_2_)),^[36]^ enabling the identification of weak intermolecular and intramolecular interactions, as illustrated in Figure 3. In this context, green and red spikes on the positive scale of sign(λ_2_) represent van der Waals interactions and steric repulsions, respectively.

The non‐covalent interactions of the investigated complex are depicted in Figure 3(A & B) at a threshold of 0.025 a.u. For the first predicted structure, the green‐colored RDG regions correspond to the interactions between the following bonds: C38⋅⋅⋅H53, C27⋅⋅⋅H55, C36⋅⋅⋅C46, C26⋅⋅⋅H51, C38⋅⋅⋅N68, H6⋅⋅⋅C26, H17⋅⋅⋅C38, C24⋅⋅⋅H69, and H57⋅⋅⋅O66.

Additionally, the NCI plot in (Figure 3B) highlights the van der Waals interactions and steric effects within the investigated complex. Notably, the aromatic moieties in the complex molecules appear to engage in van der Waals contacts, as evidenced by the strong green RDG spikes observed between the molecules in (Figure 3A). The blue dot in (Figure 3A) corresponds to a boron‐carbon bond, not to an intermolecular interaction.

#### 2.3.4 Quantum Theory of Atoms in Molecules (QTAIM)

**Figure 4:**
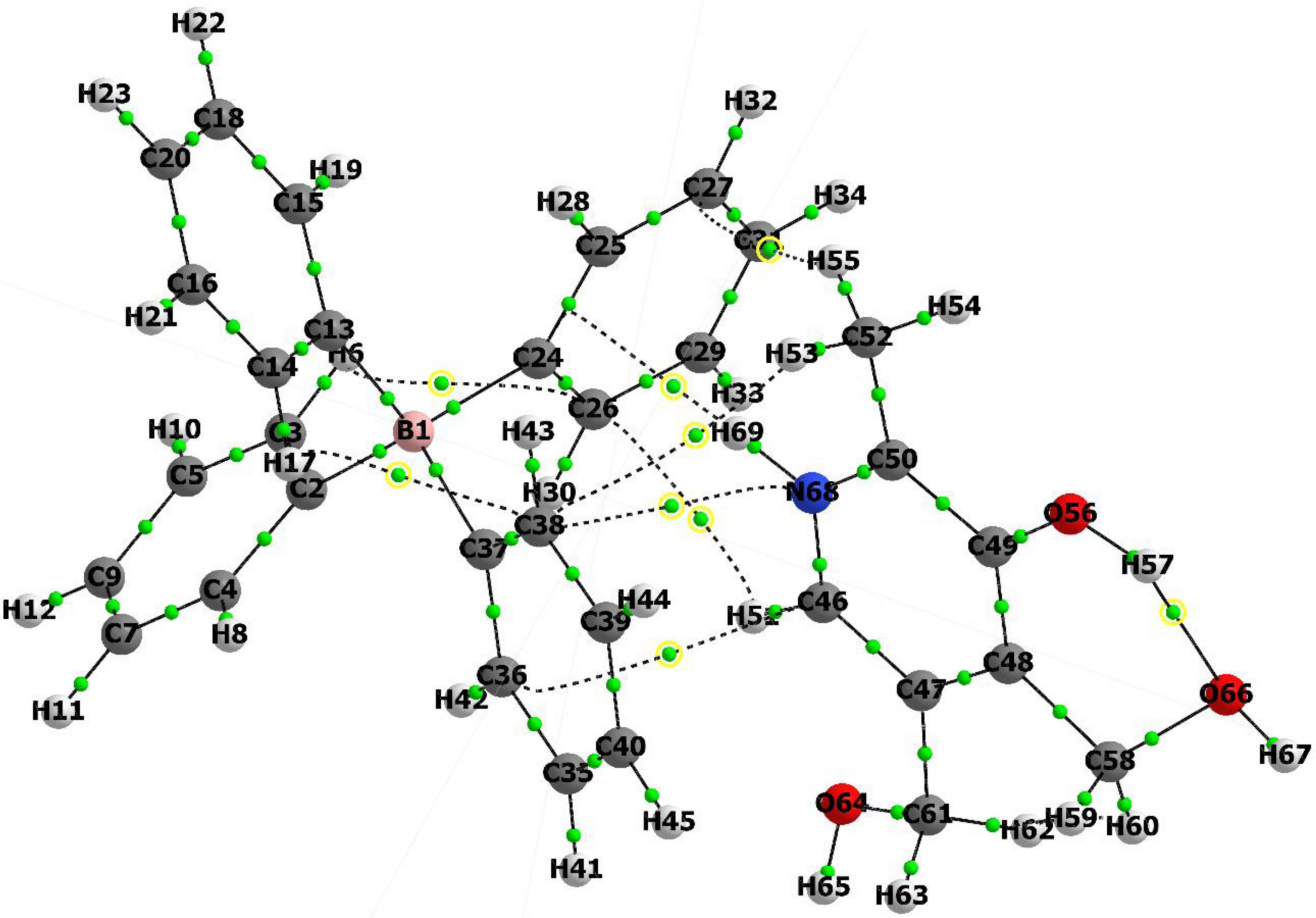
Topological atoms in molecules (AIM) graph of the both PY‐TPB complexes.

Based on the provided bond critical point (BCP) data and the Rozas et al.^[37]^ classification criteria, the hydrogen bond interactions in the studied system can be interpreted as follows:

The majority of the analyzed bonds, including C38⋅⋅⋅H53 (BCP 59), C27⋅⋅⋅H55 (BCP 58), C36⋅⋅⋅C46 (BCP 54), C26⋅⋅⋅H51 (BCP 53), C38⋅⋅⋅N68 (BCP 50), H6⋅⋅⋅C26 (BCP 28), H17⋅⋅⋅C38 (BCP 42), and C24⋅⋅⋅H69 (BCP 30), are classified as weak hydrogen bonds. This classification is based on their positive values for both the Laplacian of electron density (∇^2^ρ(r)) and the total energy density (H(r)) at their respective BCPs (Table 3). In contrast, the bond H57⋅⋅⋅O66 (BCP 71) stands out as a moderate hydrogen bond (Figure 4). This bond exhibits a positive ∇^2^ρ(r) value coupled with a negative H(r) value, indicating a stronger interaction compared to the weak hydrogen bonds (Table 3). Furthermore, all the analyzed bonds, including the moderate hydrogen bond, have |V(r)|/G(r) ratios less than 1. This signifies that these interactions fall under the category of closed‐shell interactions, suggesting a lack of significant covalent character.

**Table 3:** AIM parameters of chosen H‐bonds at bond critical points (BCPs) for the interaction between PY‐TPB complex.

BCP	bond	ρ(r) (a.u.)	V(r) (a.u.)	H(r) (a.u.)	ϵ (a.u.)	∇^2^ρ(r) (a.u.)	G(r) (a.u.)	|V(r)|/G(r)	$\frac{H\left(r\right)\;}{\rho \left(r\right)}$
59	C38⋅⋅⋅H53	0.003	−0.002	0.001	1.654	0.01	0.002	0.758	0.147
58	C27⋅⋅⋅H55	0.006	−0.003	0.001	0.892	0.016	0.003	0.815	0.098
54	C36⋅⋅⋅C46	0.006	−0.003	0.001	2.189	0.018	0.004	0.735	0.157
53	C26⋅⋅⋅H51	0.006	−0.003	0.001	0.186	0.019	0.004	0.782	0.129
50	C38⋅⋅⋅N68	0.007	−0.003	0.001	2.205	0.021	0.004	0.796	0.128
28	H6⋅⋅⋅C26	0.009	−0.005	0.002	6.946	0.035	0.007	0.738	0.202
42	H17⋅⋅⋅C38	0.009	−0.005	0.002	2.392	0.036	0.007	0.746	0.195
30	C24⋅⋅⋅H69	0.014	−0.006	0.002	1.9	0.04	0.008	0.778	0.125
71	H57⋅⋅⋅O66	0.04	−0.037	‐0.002	0.037	0.137	0.036	1.045	−0.041

#### 2.3.5 Chemical Reactivity Study (The Global Reactivity Descriptors)

Global reactivity descriptors provide valuable insights into the formation of the PY‐TPB complex, specifically the interaction between the PY cation and the TPB anion. By analyzing the reactivity profiles of both the cation and anion, we can elucidate the key driving forces behind complex formation.

As shown in Table 4, TPB exhibits a strong electrophilic character, evidenced by its high electrophilicity index (ω) and electroaccepting power (ω^+^). Conversely, PY, in its cationic form (PY+), displays weak electrophilicity and a tendency towards electron donation, as indicated by its low ω and moderate ω‐ values. This difference in reactivity, with TPB^−^ acting as an electron acceptor and PY^+^ as an electron donor, strongly suggests an interaction where electron density is transferred from PY^+^ to TPB^−^. This type of interaction aligns with the principles of charge‐transfer complexation, where an electron‐rich species (donor) interacts favorably with an electron‐deficient species (acceptor). Therefore, the global reactivity descriptors highlight the complementary nature of TPB^−^ and PY^+^ in terms of their electronic properties. This complementarity, with TPB's strong electron‐accepting capacity and PY^+^′s electron‐donating ability, emerges as a significant driving force for the formation of the PY‐TPB complex.^[38]^

**Table 4:** Global Reactivity Descriptors of PY Cation and TPB Anion and Their Implications for PY‐TPB Complex Formation.

Global reactivity descriptors	TPB	PY	PY^+^	TPB‐PY
E_HOMO_ (N) (eV)	−5.72	−5.99	−2.17	−4.92
E_HOMO_ (N+1) (eV)	−2.43	2.67	3.19	0.39
E_HOMO_ (N‐1) (eV)	−9.47	−11.65	−10.80	−8.57
Vertical IP (eV)	7.50	7.95	4.09	6.32
Vertical EA (eV)	3.73	−0.99	−1.67	1.26
Mulliken electronegativity (eV)	5.62	3.48	1.21	3.79
Chemical potential (eV)	−5.62	−3.48	−1.21	−3.79
Hardness (=fundamental gap) (eV)	3.77	8.95	5.76	5.06
Softness (eV^−1^)	0.27	0.11	0.17	0.19
Electrophilicity index (eV)	4.18	0.68	0.13	1.42
Nucleophilicity index (eV)	3.40	3.13	6.95	4.20
Electroaccepting Power (ω+) (eV)	5.79	0.17	0.01	1.26
Electrodonating Power (ω−) (eV)	11.41	3.65	1.22	5.05

#### 2.3.6 Molecular Electrostatic Nature of Interactions (MESP)

Molecular electrostatic potential (MESP) analysis provides a robust framework for understanding charge distribution and interaction dynamics within the PY‐TPB complex.^[16b]^ MESP calculations reveal localized regions of electron density accumulation and depletion, enabling the prediction of intermolecular interactions.^[16c]^

**Figure 5:**
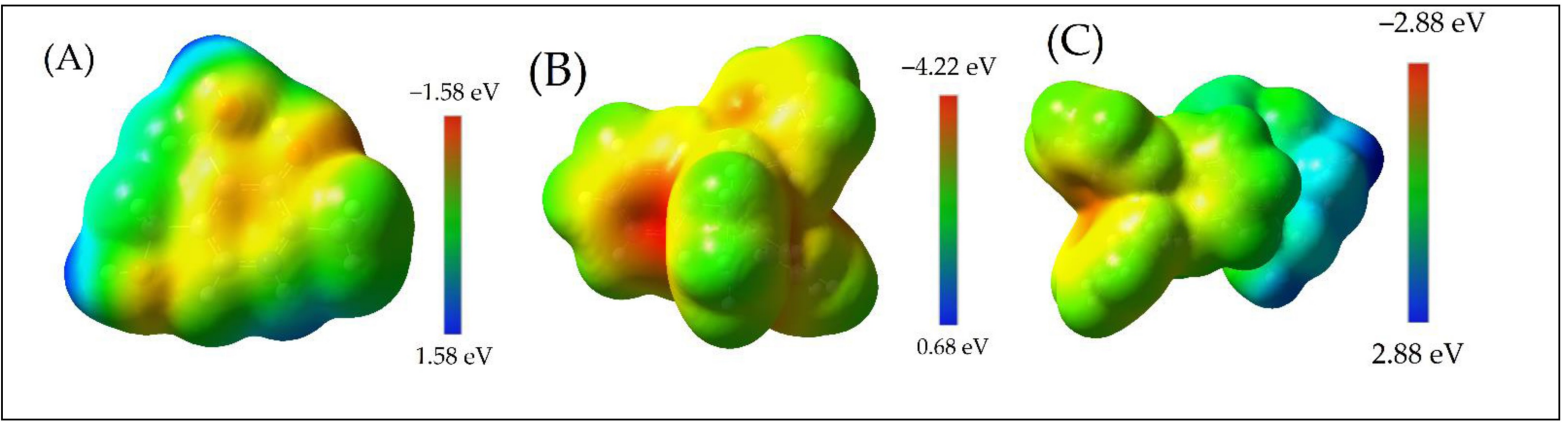
Molecular electrostatic potential maps of (A) TPB anion, (B) PY cation, and (C) PY‐TPB complex.

The TPB anion exhibits a strong negative MESP (−4.22 eV) localized around atoms proximate to the borate center (Figure 5A), indicating an electron‐rich region. Conversely, the PY cation displays positive MESP values (1.58 eV) concentrated around the amine and hydroxyl hydrogens (Figure 5B), signifying electron deficiency. These complementary electrostatic potentials facilitate the formation of the PY‐TPB complex, as evidenced by the close proximity of electron‐rich (red) and electron‐deficient (blue) regions within the complex's MESP map (Figure 5C).

The MESP values within the PY‐TPB complex range from −2.88 to 2.88 eV, underscoring the significant electrostatic interactions between the two molecules. While this analysis highlights the role of electrostatic complementarity in complex stability, further investigation into specific intermolecular interactions, such as hydrogen bonding^[39]^ and van der Waals forces,^[40]^ along with a detailed atomic charge distribution analysis, are warranted to provide a more comprehensive understanding of the complex's stability and potential applications.[^38^, ^41^]

#### 2.3.7 Analysis of Frontier Molecular Orbitals, and UV Spectra

**Figure 6:**
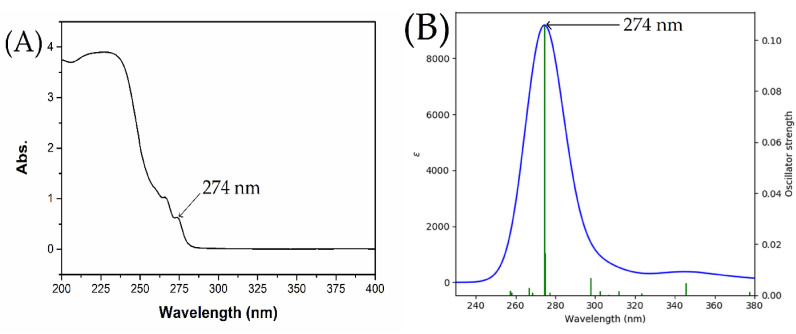
(A) Experimental absorption spectrum of the complex in water at room. (B) temperature TD‐DFT calculated absorption spectrum of the complex in water.

**Figure 7:**
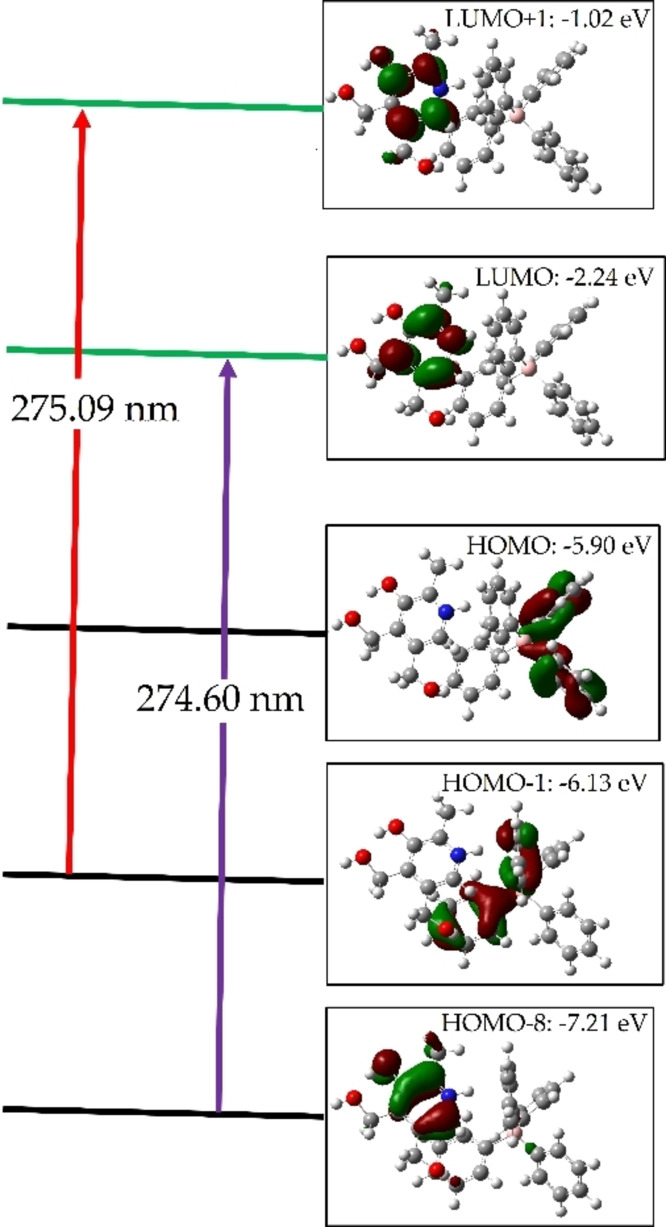
Frontier molecular orbitals (MOs) of the PY‐TPB complex in water, illustrating the orbitals involved in electronic absorption and the corresponding energy gap (ΔE).

Time‐dependent density functional theory (TD‐DFT) calculations were performed to generate molecular orbital (MO) energy diagrams for the PY‐TPB complex (Figure 6A&B and Figure 7).^[42]^ Analysis of these diagrams reveals that the highest occupied molecular orbital (HOMO) of the complex receives the greatest contributions from the HOMO‐1 and HOMO‐8 orbitals (Table 5), which are localized primarily on the phenyl ring of TPB and the pyridin‐3‐ol ring of PY, respectively. Conversely, all lowest unoccupied molecular orbitals (LUMOs) of the complex are predominantly formed from atomic orbitals of the ligands. While the LUMOs of the PY‐TPB complex exhibit contributions from the pyridin‐3‐ol ring, the LUMOs are primarily localized on the pyridin‐3‐ol moiety of PY. The calculated HOMO‐LUMO energy gap for the PY‐TPB complex is 3.63 eV.[^41^, ^43^]

**Table 5:** Calculated Electronic Properties of the PY‐TPB Complex in Water: Energy, Dipole Moment, Absorption Wavelength, Excitation Energy, Oscillator Strength, Electronic Transitions, and Primary Contributions.

Solvent	E_Total_ (a.u)	Dipole Moment	λ_max_	ƒ	Transition Energy (eV)	Electronic Transition	Major % Contribution
Water	36351.41	0.436230	275.09	0.0165	4.5070	H‐1→L+1	96
	36416.73		274.60	0.1056	4.5151	H‐8→LUMO	91

## 3 Conclusions

The proposed ion‐pair complex was formed by reacting sodium tetraphenyl borate with pyridoxine hydrochloride at room temperature. The structure of the examined complex was confirmed using several instrumental techniques, and the results indicate the structure of the complex. The examined ion‐pair complex showed weak antibacterial action against gram‐negative bacteria but showed antibacterial activity against gram‐positive bacteria and antifungal activity.

The computational investigation utilizing Density Functional Theory (DFT) provided valuable insights into the structural and electronic properties of the pyridoxine‐tetraphenylborate ion‐pair complex, complementing the experimental findings. The optimized geometries obtained at the B3LYP/6‐311G(d,p) level of theory revealed a thermodynamically favorable complex formation, as indicated by the negative complexation energies. This finding aligns with the successful experimental synthesis of the complex. Furthermore, the computational analysis shed light on the key intermolecular interactions responsible for the stability of the complex. The Reduced Density Gradient (RDG) analysis, coupled with Non‐Covalent Interaction (NCI) plots, highlighted the significant contribution of van der Waals forces in holding the pyridoxine cation and tetraphenylborate anion together. This observation suggests that the large, aromatic tetraphenylborate anion effectively interacts with the pyridoxine cation through these dispersive forces. Additionally, Quantum Theory of Atoms in Molecules (QTAIM) analysis identified the presence of both weak and moderate hydrogen bonds within the complex. These hydrogen bonds, characterized by their respective bond critical point (BCP) properties, further contribute to the overall stability of the ion‐pair. The identification of these specific interactions provides a deeper understanding of the complex's structural features.

## Conflict of Interests

The authors declare no conflict of interest.

4

## Supporting information

As a service to our authors and readers, this journal provides supporting information supplied by the authors. Such materials are peer reviewed and may be re‐organized for online delivery, but are not copy‐edited or typeset. Technical support issues arising from supporting information (other than missing files) should be addressed to the authors.

Supporting Information

## Data Availability

The data that support the findings of this study are available from the corresponding author upon reasonable request.
