# Supplementary material for: Characterization, Antibacterial Evaluation and Computational Study of Synthesized 4,5‐bis(Hydroxymethyl)‐2‐Methylpyridin‐3‐ol Tetraphenylborate Ion‐Pair Complex
Source: ChemistryOpen. 2025 Feb 10;14(3):e202400422. doi: 10.1002/open.202400422 (PMC12128165; doi:10.1002/open.202400422)
Supplement: Supplementary file 1 — Supporting Information [file OPEN-14-e202400422-s001.pdf]

ChemistryOpen

Supporting Information

Characterization, Antibacterial Evaluation and Computational Study of Synthesized 4,5-bis(Hydroxymethyl)-2-Methylpyridin-3-ol Tetraphenylborate Ion-Pair Complex

Ahmed H. Bakheit,* Mohamed H. Al-Agamy, Rashad Al-Salahi, Essam Ali, Haitham Alrabiah, and Gamal A.E. Mostafa*

Characterization, Antibacterial Evaluation and Computational study of synthesized 4,5-bis(hydroxymethyl)-2-methylpyridin-3-ol tetraphenylborate Ion-pair Complex

Ahmed H. Bakheit, [1]* Mohamed H. Al-Agamy, [2] Rashad Al-Salahi, [1] Essam A. Ali , Haitham Alrabiah,[1] and Gamal A. E. Mostafa [1]*

[1]Department of Pharmaceutical Chemistry, College of Pharmacy, King Saud University, P.O. Box 2457, Riyadh 11451, Saudi Arabia
[2] Department of Pharmaceutics, College of Pharmacy, King Saud University, P.O. Box 2457, Riyadh 11451, Saudi Arabia

* Correspondence: abakheit@ksu.edu.sa and gmostafa@ksu.edu.sa

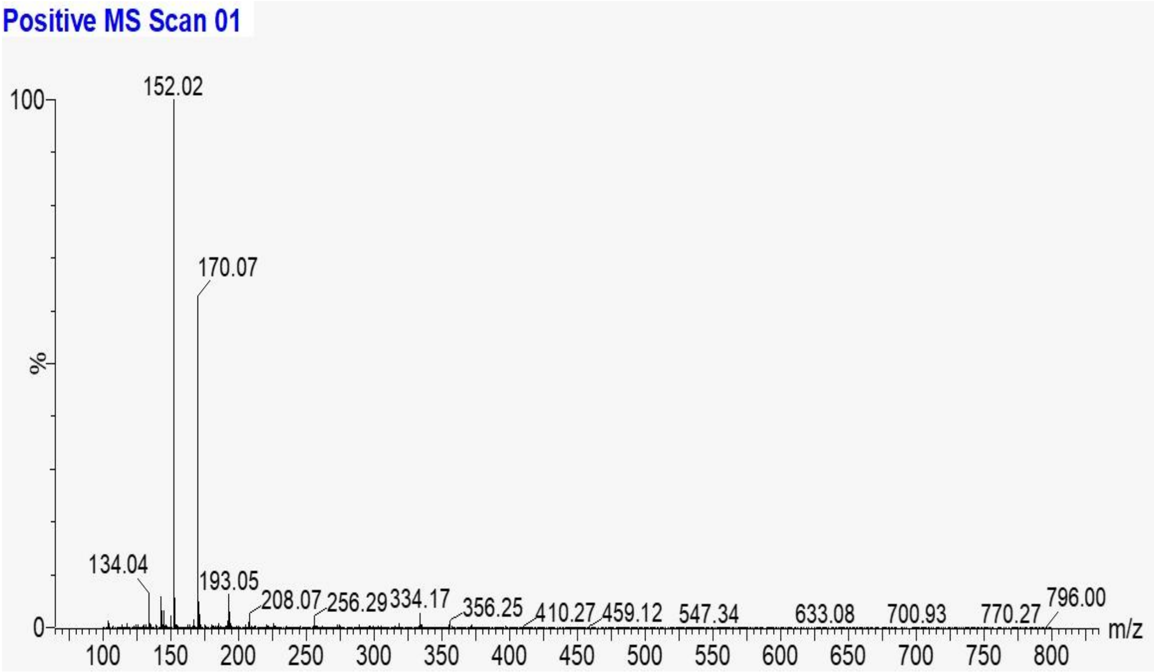

Figure S1A. Positive scan of PY-TPB complex.

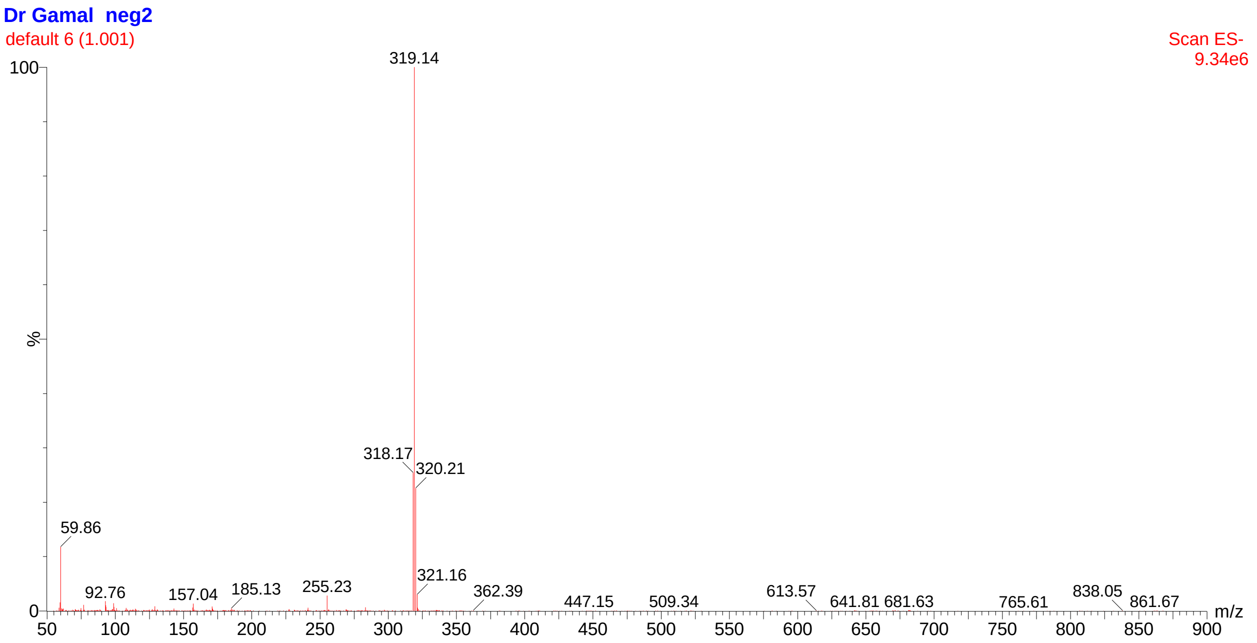

Figure S1B. Negative scan of PY-TPB complex.

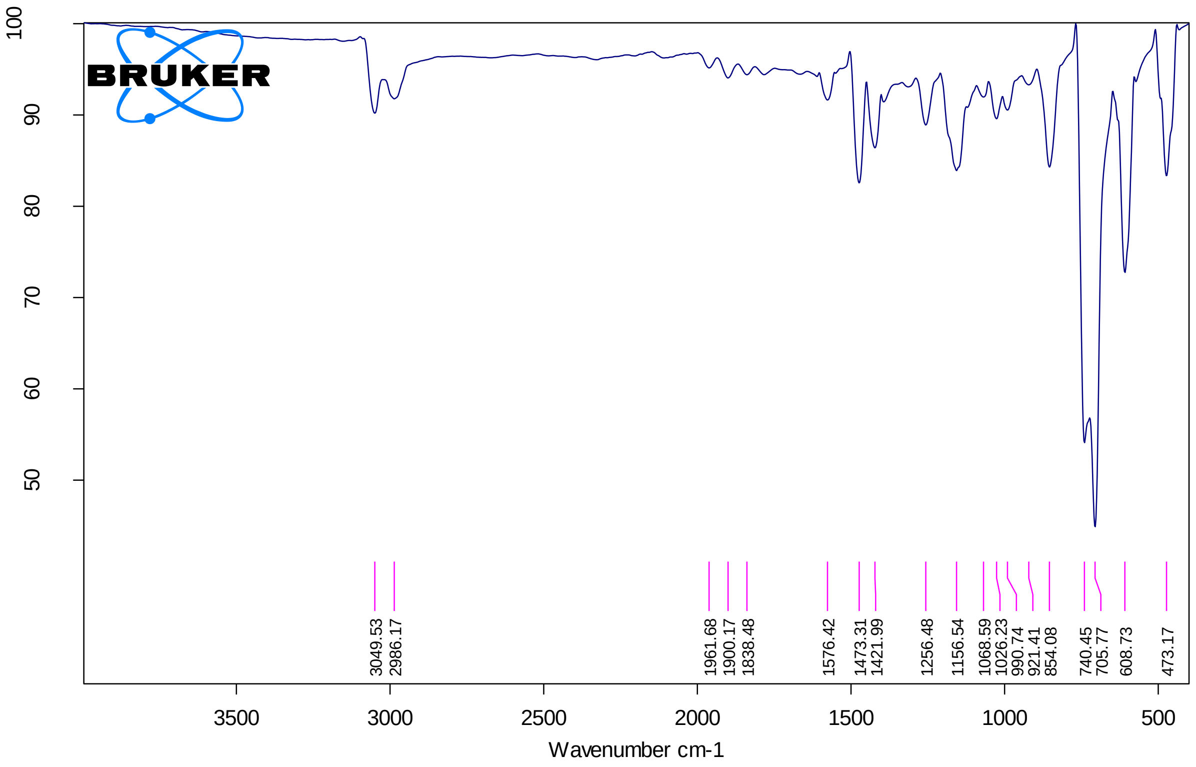

C:\Users\DELL\Documents\Bruker\OPUS_7.8.38\DATA\MEAS\TPB.0	TPB	Instrument type and / or accessory	5/23/2023

Page 1/1

Figure S2A. IR spectrum of sodium tetraphenyl borate.

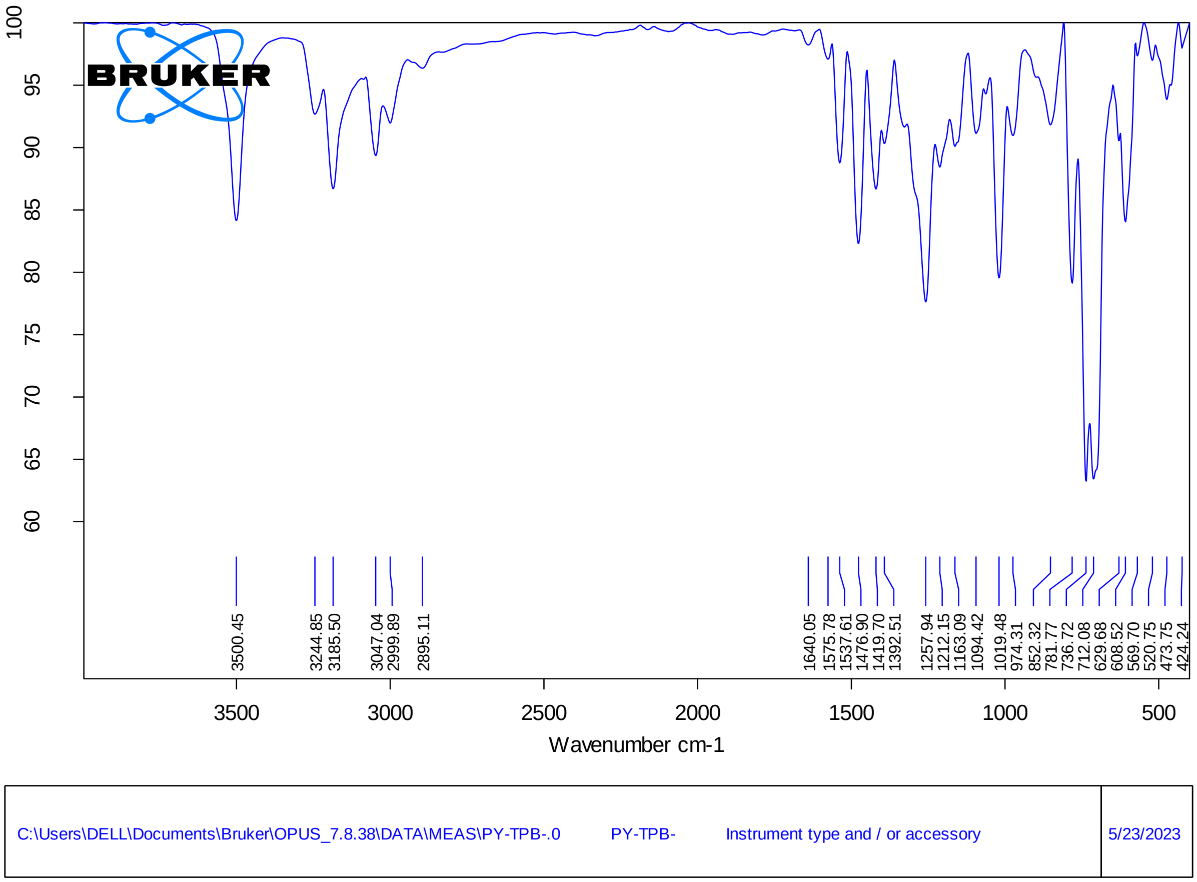

Page 1/1

Figure S2B. IR spectrum of Pyridoxine-tetraphenyl borate complex.

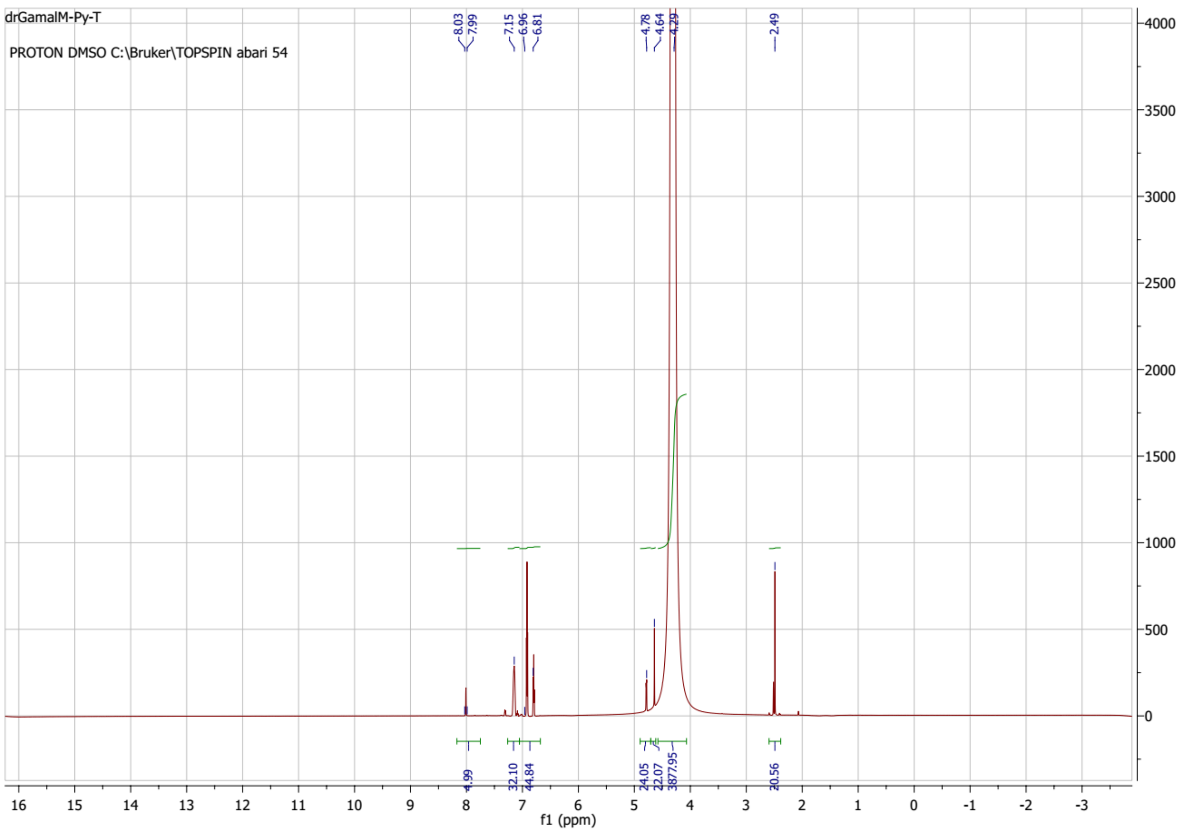

Figure S3. ^{1}H-NMR of PY-TPB complex.

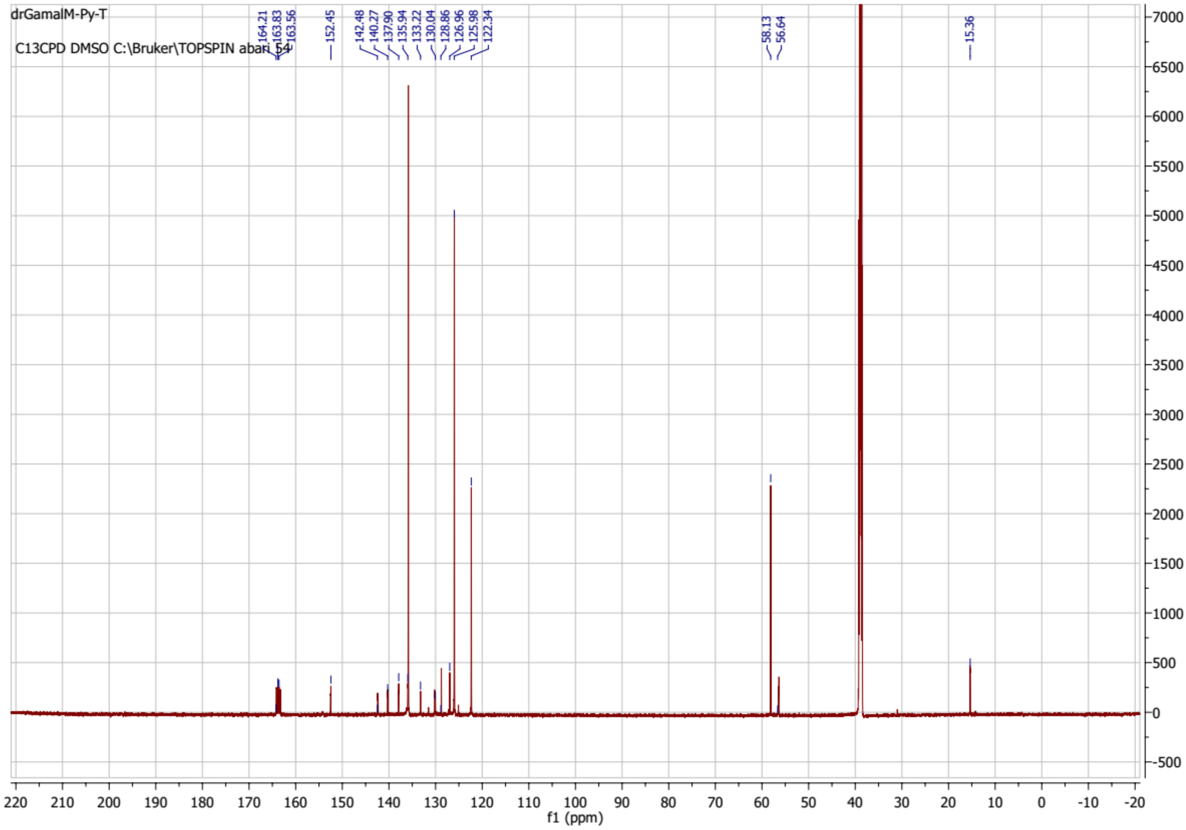

Figure S4. ^{13}C NMR of PY-TPB complex.

Table S1: Summarizing XRD peak positions, Full Width at Half Maximum (FWHM), and relative intensities (100.*I/Imax) for 4,5-bis(hydroxymethyl)-2-methylpyridin-3-ol tetraphenylborate complex.

	Peak positions			
No.	2theta	d	FWHM	100.*I/Imax
1)	10.3	8.581218	0.5182	12.24
2)	11.6	7.622285	0.3605	37.88
3)	15.5	5.712085	0.857	3.12
4)	16.6	5.335968	0.5078	1.64
5)	17.5	5.06352	0.3202	15.56
6)	20.4	4.349782	0.3505	59.05
7)	20.9	4.246835	1.5461	3.65
8)	21.6	4.110762	1.0112	5.06
9)	23.5	3.782525	0.3464	8.47
10)	26.9	3.311654	0.6651	2.2
11)	27.5	3.240747	0.6842	10.12
12)	28	3.184003	0.8839	2.09
13)	30.1	2.966473	0.8637	1.86
14)	31.3	2.855424	0.3626	4.02
15)	32.7	2.7363	0.5403	1.87
16)	38	2.365956	0.3061	100
17)	44.2	2.047396	0.3183	35.9
18)	64.5	1.443514	0.4197	6.75
19)	77.5	1.23063	0.4569	18.37
20)	81.7	1.177652	0.4473	7.08
21)	98.1	1.019858	0.4969	2.17
22)	110.9	0.935224	0.46	5.67
23)	115.3	0.911795	0.4482	4.12
24)	135.7	0.831656	0.5583	5.83
25)	136.4	0.829609	0.5366	2.09
